# Sister Mary Joseph's Nodule From Renal Cell Carcinoma: A Case Report

**DOI:** 10.7759/cureus.30344

**Published:** 2022-10-16

**Authors:** Kimiko Hirata, Masaru Narabayashi, Takaya Murashima, Takehiko Segawa, Shuji Ohtsu

**Affiliations:** 1 Radiation Oncology, Kyoto City Hospital, Kyoto, JPN; 2 Urology, Kyoto City Hospital, Kyoto, JPN

**Keywords:** umbilical skin metastasis, renal cell carcinoma, interstitial brachytherapy, palliative radiotherapy, sister mary joseph’s nodule

## Abstract

Sister Mary Joseph’s nodules (SMJNs) are umbilical skin metastases of various abdominopelvic malignancies, and they rarely originate from renal cell carcinomas. Radiotherapy is typically used to treat the nodules as a palliative intention. We report a rare case of SMJN that originated from clear cell renal cell carcinoma, which was treated with external beam radiation therapy (EBRT) and interstitial brachytherapy (ISBT).

A 74-year-old male patient with a history of left renal cell carcinoma developed an umbilical nodule which was diagnosed as SMJN. The patient underwent EBRT (30 Gy in 10 fractions) and ISBT (12 Gy in two fractions), leading the nodule to complete resolution. This case report might support that radiotherapy, including ISBT, is effective for the treatment of SMJN from renal cell carcinoma.

## Introduction

Sister Mary Joseph’s nodules (SMJNs) are umbilical skin metastases of various abdominopelvic malignancies. They can originate from gastric, colonic, pancreatic, and ovarian cancers [1]. Although their occurrence in abdominopelvic malignancies is reported to be 1% to 3% [2], SMJNs from renal cell carcinoma are exceedingly rare, with only a few documented cases in the literature [3].

No consensus exists on the treatment for SMJN, even though studies have reported successful surgical resection [4]. The nodules can be painful or exudative; thus, patients often seek palliative treatment. Although radiotherapy is occasionally used in clinical practice, palliative radiotherapy for SMJN has not been reported in many studies [5]. We describe a case of SMJN from renal cell carcinoma that was successfully treated with external beam radiation therapy (EBRT) and interstitial brachytherapy (ISBT).

This article was previously presented as an abstract at the 34th Annual Meeting of the Japanese Society for Radiation Oncology on November 12, 2021.

## Case presentation

**Figure 1:**
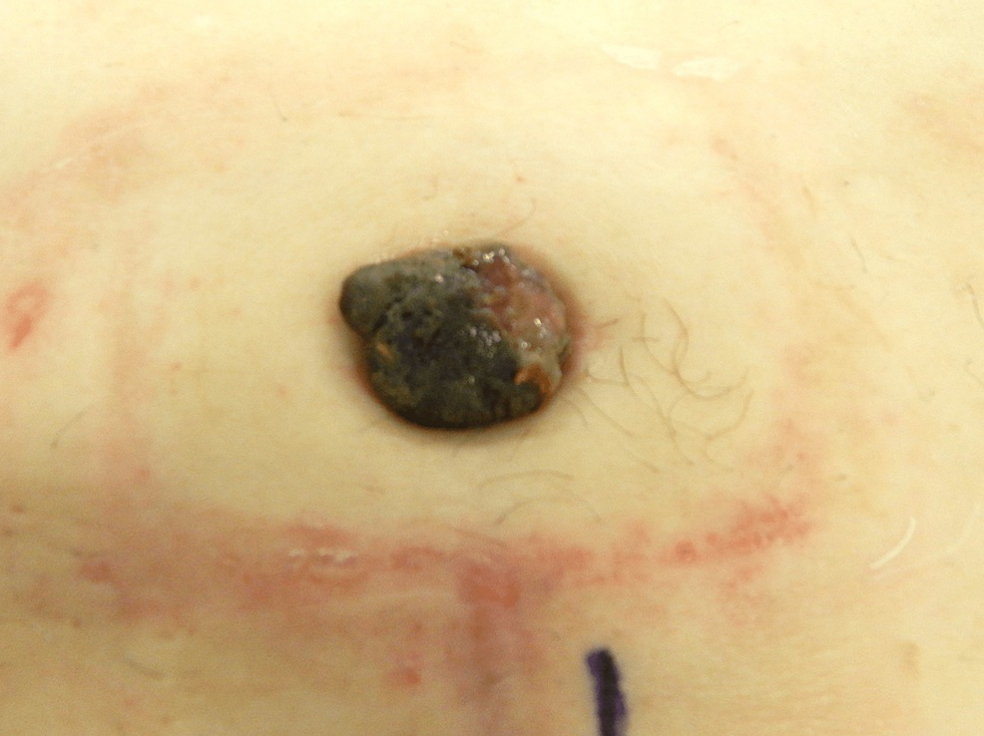
Picture of Sister Mary Joseph’s nodule (SMJN) The picture taken six months after the diagnosis of SMJN shows the umbilical nodule which was black and soft measuring approximately 30 mm.

**Figure 2:**
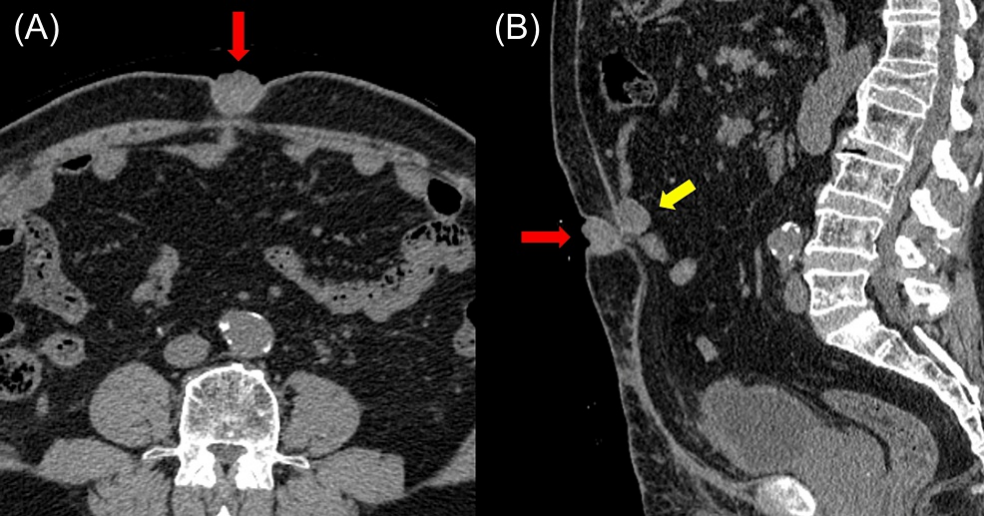
Computed tomography (CT) images of Sister Mary Joseph’s nodule The CT images show the umbilical nodule in (A) and (B) marked by red arrows. A peritoneal dissemination nodule is also depicted in (B) marked by the yellow arrow.

A 74-year-old man with a history of left clear cell renal cell carcinoma (pathological diagnosis pT1b) developed an umbilical nodule three years post a partial left nephrectomy . The patient’s medical history was right ureteral cancer, type 2 diabetes, and chronic renal failure. He had no family medical history. Significant results of laboratory analysis included red blood cell count of 398 × 104/uL (430-560 × 104/uL), hemoglobin 12.2 g/dL (13-17 g/dL), hematocrit 36.1% (39%-53%), urea nitrogen of 33.9 mg/dL (8-21 mg/dL), creatinine of 2.4 mg/dL (0.3-1.1 mg/dL), blood glucose of 112 mg/dL (70-110 mg/dL), and hemoglobin A1c of 6.6 % (4.6%-6.2%). On physical examination, the umbilical nodule was 15 mm in size, bright red, and hemorrhagic. A biopsy of the umbilical nodule confirmed the diagnosis of metastatic clear cell renal cell carcinoma. Computed tomography (CT) scan 34 months after the partial left nephrectomy revealed a 12 × 18 × 14 mm umbilical nodule, peritoneal dissemination, and a solitary pulmonary nodule, which was highly suggestive of metastasis. The patient received pazopanib administration of 200 mg once daily and maintained at 600 mg once daily, but the nodule was unresponsive to treatment as evidenced by increasing growth, exudate, malodor, and spontaneous pain. We attempted to apply the Mohs paste to the nodule and ligate the nodule; however, these attempts were ultimately ineffective and the nodule progressed gradually. Surgical resection was considered, however, surgeons did not opt for it owing to a high possibility of local recurrence for two reasons. First, resecting the nodule with adequate margins was considered to be particularly difficult; second, they could not dismiss the possibility of another recurrent umbilical lesion after the surgical resection because peritoneal dissemination was considered to be one of the potential origins of the nodule. Therefore, the patient presented to the radiotherapy department six months after the diagnosis of SMJN. Physical examination at the radiotherapy department revealed a 30-mm black and soft umbilical nodule (Figure 1), with rich vascularity on the ultrasonography image. The CT scan conducted six months after the diagnosis of SMJN revealed that the umbilical nodule was now 28 × 24 × 27 mm in size (Figure 2) and the peritoneal dissemination placed immediately dorsal to the umbilical nodule was 20 × 22 × 25 mm in size (Figure 2 (B)).

The EBRT was conducted using a linear accelerator with a 10-MV photon beam to decrease the blood flow to the nodule. A dose of 30 Gy in 10 fractions was delivered to the umbilical tumor and a nearby peritoneal dissemination nodule. The nodule’s size decreased by 10 mm after EBRT, with a concomitant decrease in malodor, pain, and exudate.

**Figure 3:**
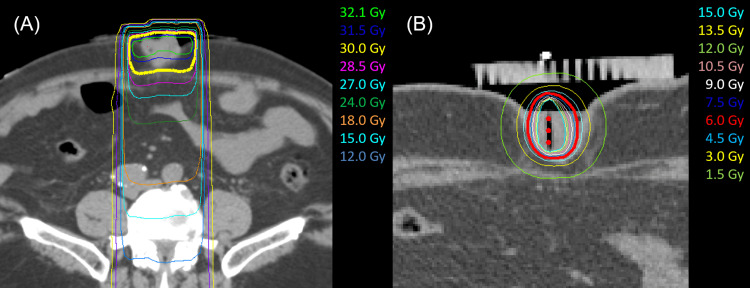
Treatment plan for radiotherapy The dosage distribution of external beam irradiation (A) and that of the first interstitial brachytherapy (B).

The CT-guided ISBT was subsequently initiated one week after EBRT. Two needles were inserted into the nodule under ultrasound guidance during the first ISBT, and a single needle was inserted during the second ISBT. The patient was treated using a high-dose-rate Ir-192 remote afterloading system. The total dose of ISBT was 12 Gy administered weekly in two fractions. The CT images from EBRT and the first ISBT are shown in Figure 3. Doses delivered to 90% of the clinical target volume (CTV) were 31.2 Gy, 6.5 Gy, and 5.8 Gy for EBRT, the first ISBT, and the second ISBT, respectively; the mean doses to the CTV were 32.2 Gy, 16.9 Gy, and 15.7 Gy for EBRT, the first ISBT, and the second ISBT, respectively.

**Figure 4:**
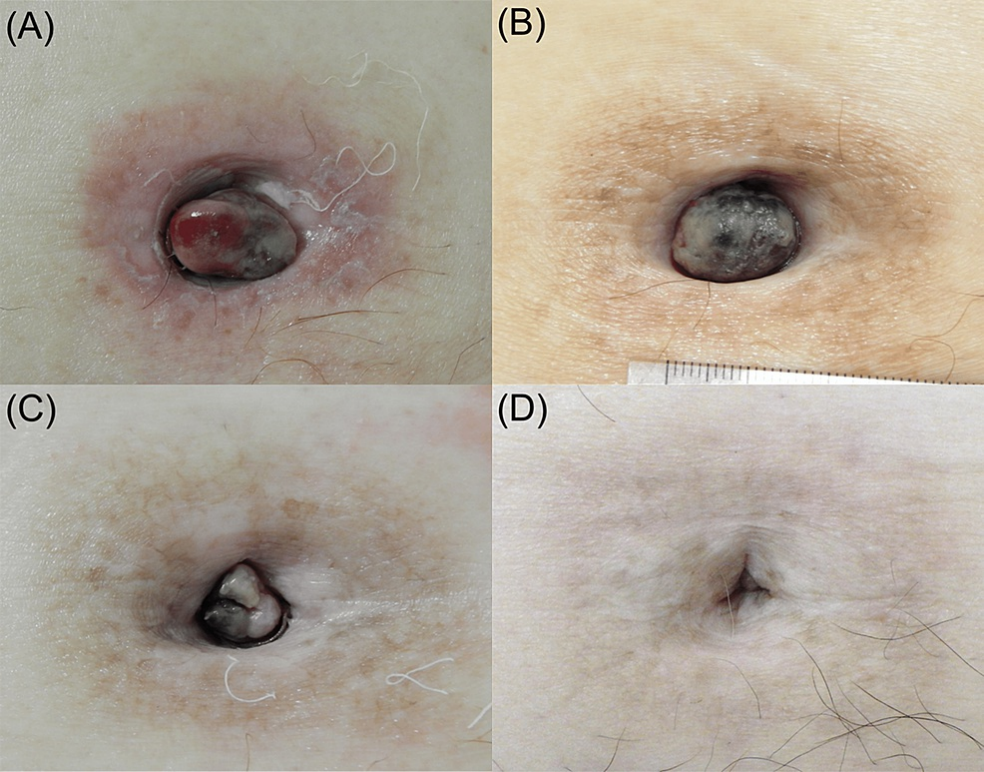
Pictures showing changes in Sister Mary Joseph’s nodule The umbilical nodule at 10 days (A), one month (B), two months (C), and four months (D) after the second interstitial brachytherapy.

No complications were observed from the needle insertion. The patient developed grade 1 radiation dermatitis one week after the second ISBT (Common Terminology Criteria for Adverse Events version five). After ISBT, the nodule progressively decreased in size until complete resolution at five months (Figure 4). The exudate from the nodule resolved two months after ISBT. At the two-year follow-up, which included a visual and palpation examination every two months and a CT scan every six months, the patient did not present with any SMJN recurrence.

The patient continued to take pazopanib for the other metastatic sites for 15 months; however, the course was switched to nivolumab as the pulmonary metastasis continued to grow. He stopped the course of nivolumab after taking it for three months because of severe systemic skin toxicity and received no further treatment. He now lives with asymptomatic peritoneal and pulmonary disease.

## Discussion

We report a rare case of SMJN that originated from clear cell renal cell carcinoma, which was successfully treated with radiotherapy. To the best of our knowledge, this represents the first documented report of ISBT for SMJN.

Sister Mary Joseph’s nodules usually originate from abdominopelvic malignancies. Hugen et al. reported that SMJNs most frequently originated from colonic (43.8%), gastric (10.5%), and pancreatic (10.5%) malignancies in male patients and from ovarian (38.8%) and endometrial (7.6%) malignancies in female patients [1]. Moreover, according to Hugen et al., only 2.9% SMJNs originated from prostate cancers, and SMJNs from urothelial or renal origins were not reported. Chen et al. reported the only known case report of SMJN from renal primary; they described a case of renal cell carcinoma with peritoneal metastases and an SMJN [3]. The possible mechanism for this is that renal cell carcinoma typically spreads via extra-renal extension, lymphatic dissemination, or venous invasion by the tumor and intraperitoneal spread may occur as a result of disruption of the renal capsule [4].

Patients with an SMJN have a poor prognosis, with a median overall survival of 6.7 to 9.1 months [1]. The treatment of an SMJN is palliative; surgery (wide excision) and radiotherapy have proven ineffective [6]. Iavazzo et al. have reported a case of SMJN from a peritoneal primary treated with combined external beam radiotherapy and tamoxifen. However, the nodule became refractory to treatment and increased in size, resulting in wide excision of the nodule [5]. The size of SMJN typically ranges from 0.5 to 2 cm, reaching in some cases the size of 10 cm [4]. In our case, the nodule size was 3 cm and relatively small, owing to which surgery was considered. However, because of the difficulty in resecting the nodule with adequate margins and the possibility that peritoneal dissemination was one of the potential origins of the umbilical lesion, local recurrence after the surgery was considered highly likely. The European Society for Medical Oncology guideline states that radiation therapy is an effective treatment for the palliation of local and symptomatic metastatic renal cell carcinoma disease or the prevention of the progression of metastatic disease [7]. We selected radiation therapy for the patient, despite the nodule being an indication for surgery. Therefore, this case shows that radiation therapy, including ISBT, can be a treatment option for patients unsuitable for surgery or those who refuse it.

Brachytherapy can deliver targeted high-dose therapy with steep dose gradients to tumors while sparing surrounding organs and critical structures. There are several brachytherapy techniques: surface, intracavitary including intraluminal, and interstitial applications [8]. Owing to its invasiveness, ISBT is primarily used to treat cervical, prostate, breast, and several oral malignancies to cure them. Although literature reports on palliative ISBT are lacking, it is anecdotally considered useful in the palliative setting. Several studies have demonstrated the efficacy of palliative intraluminal irradiation for esophageal and non-small cell lung cancers. Homs et al. randomized 209 patients with dysphagia secondary to inoperable esophageal cancer to esophageal stenting or intraluminal brachytherapy, with superior long-term palliation with brachytherapy compared with stenting [9]. Appalanaido et al. reported a case of axillary metastasis from hypopharyngeal cancer treated with ISBT with one fraction of 6 Gy daily for nine consecutive days to relieve pain and bleeding. The patient also underwent intraluminal brachytherapy on the primary tumor for the treatment of dysphagia [10]. Our patient demonstrated favorable performance status and had no life-threatening tumor burden at the time of evaluation. These reassuring factors encouraged us to proceed with palliative ISBT.

In the present case, we performed ISBT to provide targeted high-dose radiation to the nodule while minimizing the radiation to the surrounding skin and intestines. The interstitial application is relatively easy and safe because SMJN is externally visible. The cumulative biologically effective dose (BED) given to the tumor periphery, evaluated according to the value of α/β = 3 Gy (BED3) of EBRT + ISBT was 101.2 Gy, and the center of the tumor received a higher dose due to the nature of ISBT with the cumulative mean dose to CTV being 64.8 Gy (BED3 = 276.7 Gy). De Meerleer et al. suggested several schedules for stereotactic body radiation therapy (SBRT) of renal cell carcinoma to overcome radioresistance. The SBRT can achieve an overall response rate of about 90% and low toxicity using 24 Gy in one fraction, 32 Gy in two fractions, 36 Gy in three fractions, and 35 Gy in five fractions. The BED3 of these SBRT schedules was 117-202 Gy [11]. Zelefsky et al. reported that single fractionated SBRT of 24 Gy (BED3 = 216 Gy) achieves encouraging local control compared to single fractionated SBRT of 18-22 Gy in one fraction (BED3 = 126-183 Gy) or hypo-fractionation radiotherapy of 24-30 Gy in three-five fractions (BED3 = 88-90 Gy) in the prospective follow-up of 105 patients with extracranial renal cell carcinoma metastases [12]. A recent prospective trial also showed a good disease control rate of 83% with single fractionated SBRT of 20 Gy for peripheral dose and 25 Gy for central dose (BED3 = 153 and 233 Gy) [13]. In our case, the dose in the peripheral region of CTV was comparable to the dose for curative intent and the center of the tumor received a much higher dose due to the nature of ISBT. The proper dosage of radiation for the treatment of SMJN is unknown as there are few case reports [14]. Although the high dose of ISBT may have been excessive to control SMJN, our case achieved long-term disease control and our treatment algorithm may be considered for similar patients with SMJN given the successful outcome. However, further studies are required to evaluate the proper dosage to achieve symptomatic relief and disease control.

## Conclusions

An SMJN typically originates from abdominopelvic malignancies and very rarely from renal cell carcinoma. There is no consensus on the treatment of choice, which is usually palliative, and treatment options include surgical excision and radiotherapy. Because of its invasive nature, ISBT is not generally indicated for palliative therapy.

However, ISBT for SMJN is relatively easy and safe. Here, we report a case of SMJN from renal cell carcinoma that was successfully treated with radiotherapy, including ISBT. This case suggests that radiotherapy, including ISBT, may be a suitable option for the treatment of SMJN.
